# Spontaneous Splenic Rupture Following Severe Cough in a Patient With Community-Acquired Pneumonia: A Case Report

**DOI:** 10.7759/cureus.85010

**Published:** 2025-05-29

**Authors:** Al Motasim Bella Abu Laban, Waleed Muhammad

**Affiliations:** 1 Acute Internal Medicine, Royal Surrey County Hospital, Guildford, GBR

**Keywords:** community-acquired pneumonia (cap), emergency splenectomy, internal bleeding, respiratory complications, spontaneous splenic rupture, surgical emergency

## Abstract

We describe a rare case involving the spontaneous rupture of the spleen in a man in his mid-70s who initially came to the emergency department with progressively worsening shortness of breath, a productive cough with yellow-green sputum, and chest pain worsened by breathing. He was initially diagnosed with pneumonia. However, shortly after admission, he rapidly deteriorated, developing severe abdominal pain, a sudden drop in blood pressure, and a significant decrease in hemoglobin, indicating acute internal bleeding. An urgent computed tomography (CT) scan confirmed extensive bleeding around his spleen, consistent with spontaneous rupture, likely due to increased abdominal pressure from intense coughing. The patient underwent emergency surgery to remove his spleen and evacuate blood from the abdomen, followed by care in the intensive care unit. His recovery afterward was smooth, and he gradually improved without further complications. This case highlights the need for doctors to remain alert to spontaneous splenic rupture when patients with respiratory infections develop unexplained abdominal symptoms and internal bleeding. Early diagnosis and rapid surgical treatment proved essential in ensuring a positive outcome for this patient.

## Introduction

Spontaneous splenic rupture (SSR) is an uncommon yet potentially lethal clinical emergency characterized by rupture of the spleen without prior trauma or direct external injury. Typically, SSR is linked to underlying pathologies such as infections, hematologic malignancies, inflammatory conditions, or systemic diseases that affect splenic integrity and predispose the organ to rupture [1,2]. Although respiratory infections are infrequently cited causes, vigorous coughing associated with severe respiratory illnesses, including pneumonia, can substantially elevate intra-abdominal pressure, potentially precipitating splenic rupture in susceptible patients [3,4].

Several case reports in the literature have highlighted instances of SSR triggered by severe coughing episodes. For example, Kocael et al. reported a rare case of SSR associated with pneumonia, emphasizing the need for a high index of suspicion during atypical presentations [4]. Similarly, Murarka et al. documented SSR secondary to pneumonia-induced severe coughing, underscoring the clinical importance of recognizing such rare presentations early [5]. Mackenzie and Soiza described a case where SSR mimicked pneumonia, further illustrating potential diagnostic challenges in clinical practice [6].

Despite the rarity of this complication, its rapid progression to life-threatening internal hemorrhage necessitates immediate recognition and surgical intervention. A high index of suspicion and timely diagnostic imaging remain critical in achieving favorable clinical outcomes [2,5]. In this report, we present a distinctive case of SSR following forceful coughing episodes in a patient diagnosed with community-acquired pneumonia, underscoring the importance of clinical vigilance and prompt surgical management in such atypical scenarios.

## Case presentation

A male patient in his mid-70s presented to the emergency department with an 11-day history of progressively worsening shortness of breath upon exertion, accompanied by a productive cough yielding yellow-green sputum and sharp, breathing-related chest pain. He reported intermittent feverish sensations, night sweats, and mild abdominal discomfort. There was no recent history of trauma or vomiting nor any blood in the sputum. His medical history included hypertension, high cholesterol, diverticular disease, and an episode of bilateral pneumonia many years prior.

**Figure 1:**
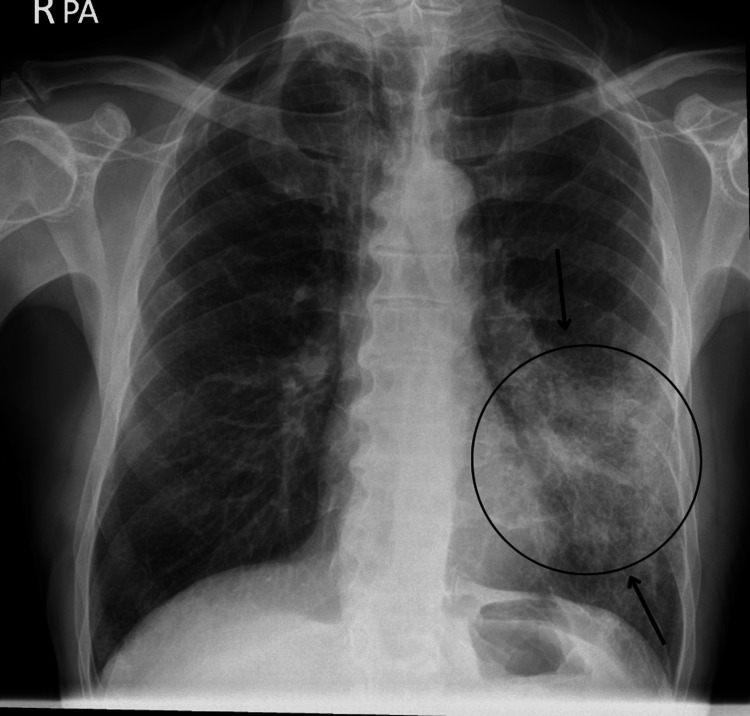
Chest X-ray at presentation showing left lower zone opacification (arrow+circle) consistent with pneumonia Black arrows and the circle indicate areas of consolidation in the left mid and lower lung zones, consistent with community-acquired pneumonia. No evidence of pleural effusion or pneumothorax is present.

Upon initial assessment, the patient's vital signs were stable. Physical examination revealed coarse crepitations predominantly in the left lower lung field. Abdominal examination at this stage revealed mild, diffuse tenderness in the left upper quadrant, without significant guarding or rigidity. Initial laboratory investigations demonstrated elevated inflammatory markers, neutrophilia, mild hyponatremia, hypokalemia, and a normal hemoglobin level (Table 1). Chest radiography revealed consolidation in the left mid and lower lung zones, consistent with community-acquired pneumonia (Figure 1). The patient was commenced on intravenous antibiotics (amoxicillin and clarithromycin).

**Table 1:** Initial laboratory investigations upon admission, with corresponding normal reference ranges Initial laboratory findings on presentation demonstrating elevated inflammatory markers, mild hyponatremia, and hypokalemia. Normal reference ranges are provided for comparison.

Test	Result	Normal range
Hemoglobin	134 g/L	130-170 g/L
White blood cell count	18.4×10⁹/L	4.0-11.0×10⁹/L
Neutrophils	16.9×10⁹/L	2.0-7.0×10⁹/L
C-reactive protein	197 mg/L	<5 mg/L
Sodium	131 mmol/L	135-145 mmol/L
Potassium	3.1 mmol/L	3.5-5.1 mmol/L

**Figure 2:**
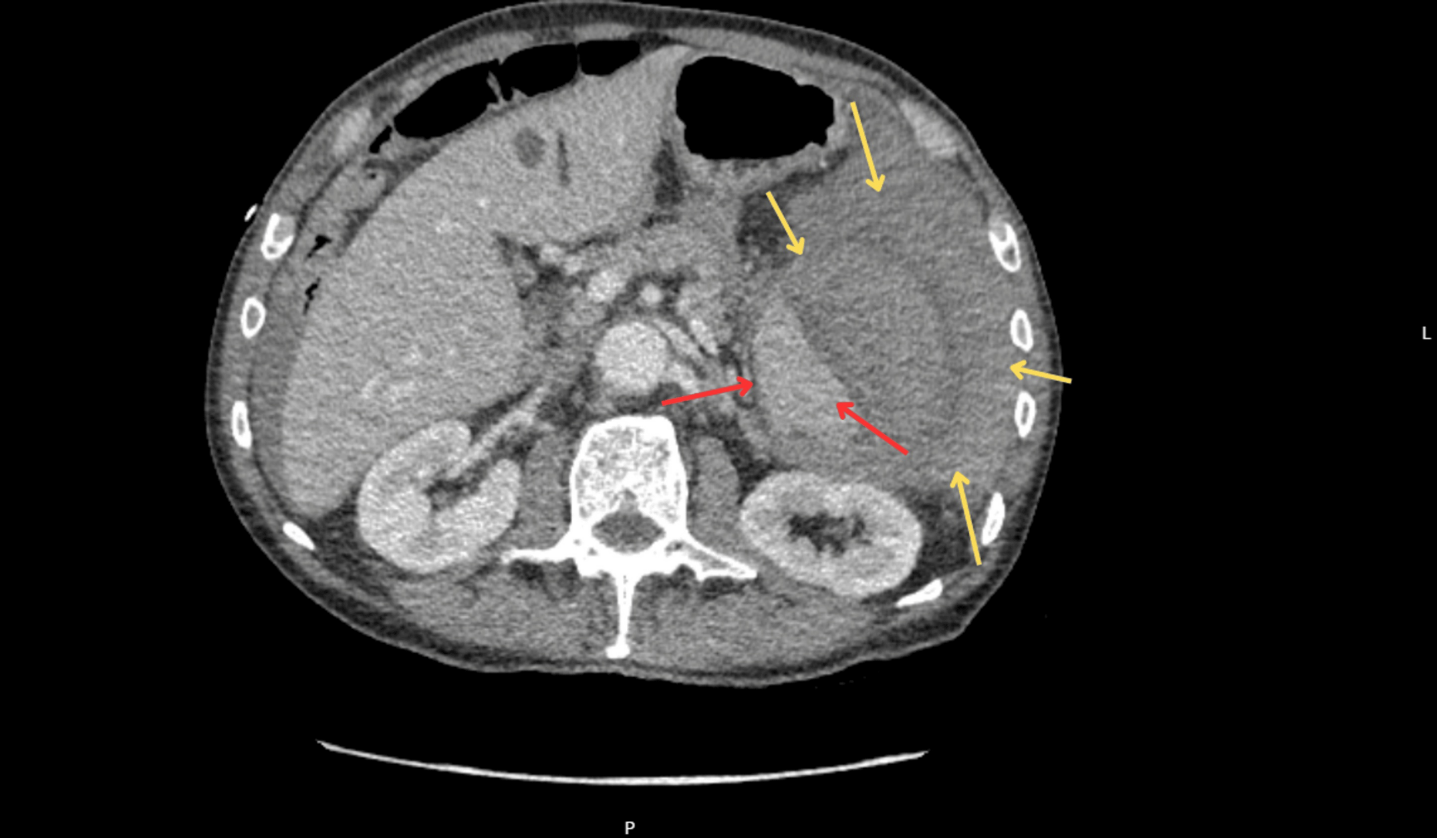
Contrast-enhanced CT of the abdomen showing the spleen (red arrow) and perisplenic fluid collection (yellow arrow), consistent with hemoperitoneum Axial CT image of the abdomen demonstrating a large perisplenic hematoma (yellow arrow) and free intra-abdominal fluid, consistent with hemoperitoneum. These findings are indicative of a spontaneous splenic rupture. CT: computed tomography

On the second day of hospitalization, the patient experienced sudden clinical deterioration, presenting with severe abdominal pain and hypotension. Laboratory investigations showed a marked decline in hemoglobin and elevated serum lactate levels, consistent with acute internal bleeding (Table 2). An urgent computed tomography (CT) scan of the abdomen and pelvis demonstrated a large acute perisplenic hematoma and extensive hemoperitoneum, indicative of SSR (Figure 2). The rupture was suspected to be secondary to increased intra-abdominal pressure caused by forceful coughing episodes.

**Table 2:** Trends in hemoglobin and serum lactate levels during clinical deterioration Laboratory findings following clinical deterioration on day 2 of admission highlighting a drop in hemoglobin and a rise in serum lactate levels, consistent with acute internal bleeding.

Parameter	Day 1	Day 2	Normal range
Hemoglobin (g/L)	134	95	130-170
Serum lactate (mmol/L)	1.6	2.8	0.5-2.2

The patient underwent emergency exploratory laparotomy, which confirmed a ruptured spleen and approximately 3.95 liters of hemoperitoneum. Splenectomy was performed, with meticulous control of bleeding at the splenic hilum using a vascular stapling device. Postoperative histological evaluation of the removed spleen confirmed rupture without underlying malignancy or significant abnormality.

The postoperative course was managed in an intensive care setting, where the patient received blood transfusions, intravenous analgesia, antibiotics, and early physiotherapy. He exhibited gradual improvement, transitioning smoothly from intensive care to a regular surgical ward. He resumed oral intake, began mobilizing independently, and was eventually discharged home. At follow-up, he described persistent mild exertional breathlessness but had successfully resumed his normal daily activities.

## Discussion

SSR remains an uncommon clinical entity, typically arising without direct trauma or external injury. It commonly occurs in patients who have underlying conditions such as hematologic malignancies, infectious diseases, or systemic inflammatory processes, which predispose the spleen to rupture due to structural fragility [1,2]. Respiratory infections, particularly those causing persistent and forceful coughing, are rare but recognized triggers. The mechanism involves repetitive and forceful cough-induced elevations in intra-abdominal pressure, potentially causing splenic capsular tearing or rupture in vulnerable patients [3].

In this reported case, the absence of a history of trauma, malignancy, or known hematologic disorders strongly suggested that severe coughing associated with community-acquired pneumonia led to SSR. While pneumonia is rarely cited as a direct cause of SSR, isolated cases documented in the literature align with the clinical scenario observed in this patient [3-5]. For instance, Kocael et al. [4] and Murarka et al. [5] described similar cases in which forceful coughing episodes associated with pneumonia led to SSR, highlighting the necessity of considering this diagnosis when patients present with unexplained sudden abdominal pain, hemodynamic instability, or acute anemia during severe respiratory infections.

While our patient's rupture resulted from cough-induced intra-abdominal stress, other rare causes, such as wandering spleen with torsion, must be considered in atraumatic rupture [6]. Both scenarios underscore the need for prompt imaging and surgical management.

Diagnosing SSR can pose significant clinical challenges, especially when symptoms overlap with the primary illness, potentially delaying timely intervention. In our patient, the rapid onset of abdominal pain accompanied by hemodynamic instability and a significant drop in hemoglobin raised immediate suspicion of internal bleeding, prompting urgent imaging. CT scanning remains the diagnostic modality of choice, accurately identifying hemoperitoneum and splenic injury, thus facilitating urgent surgical planning and intervention [1,5].

Prompt surgical management, particularly splenectomy, remains the definitive treatment for SSR, especially when substantial internal bleeding is involved. Surgical exploration allows for immediate hemorrhage control and prevents potentially fatal complications. Furthermore, this case highlights the necessity for meticulous postoperative care, including blood transfusions, appropriate antibiotic coverage, pain management, and early physiotherapy, facilitating an efficient recovery trajectory. Postoperative patient education regarding vaccinations and long-term prophylactic antibiotics to prevent overwhelming post-splenectomy infections is essential and was carefully addressed in this patient's care [1].

This case underscores critical learning points, notably the importance of heightened clinical awareness regarding SSR in patients experiencing severe cough and pneumonia. Prompt recognition of clinical signs and timely diagnostic imaging are vital steps toward achieving a favorable clinical outcome, reinforcing the value of multidisciplinary care approaches in rare but serious complications such as SSR.

## Conclusions

This case illustrates a rare but serious complication of pneumonia, SSR, likely triggered by forceful coughing. Clinicians should maintain a high index of suspicion for internal hemorrhage, especially SSR, in patients with respiratory infections who develop acute abdominal symptoms, hemodynamic instability, or a significant drop in hemoglobin. Early recognition, timely imaging, and urgent surgical intervention were critical in achieving a favorable outcome for our patient. This case emphasizes the importance of maintaining a broad differential diagnosis in complex presentations and highlights the value of multidisciplinary care in managing unexpected and potentially life-threatening complications.
